# Natural language processing for automatic evaluation of free-text answers — a feasibility study based on the European Diploma in Radiology examination

**DOI:** 10.1186/s13244-023-01507-5

**Published:** 2023-09-19

**Authors:** Fabian Stoehr, Benedikt Kämpgen, Lukas Müller, Laura Oleaga Zufiría, Vanesa Junquero, Cristina Merino, Peter Mildenberger, Roman Kloeckner

**Affiliations:** 1 Department of Diagnostic and Interventional Radiology, University Medical Center, Johannes Gutenberg-University Mainz, Langenbeckst, 1, 55131 Mainz, Germany; 2 Empolis Information Management GmbH, Leightonstraße 2, 97074 Würzburg, Germany; 3 Department of Radiology, Hospital Clínic de Barcelona, C. de Villarroel, 170, 08036 Barcelona, Spain; 4 European Board of Radiology (EBR), Barcelona, Spain; 5 Institute of Interventional Radiology, University Hospital Schleswig-Holstein, Campus Luebeck, Ratzeburger Allee 160, 23583 Luebeck, Germany

**Keywords:** Natural language processing, Free-text answers, Radiological, Education, Automatization

## Abstract

**Background:**

Written medical examinations consist of multiple-choice questions and/or free-text answers. The latter require manual evaluation and rating, which is time-consuming and potentially error-prone. We tested whether natural language processing (NLP) can be used to automatically analyze free-text answers to support the review process.

**Methods:**

The European Board of Radiology of the European Society of Radiology provided representative datasets comprising sample questions, answer keys, participant answers, and reviewer markings from European Diploma in Radiology examinations. Three free-text questions with the highest number of corresponding answers were selected: Questions 1 and 2 were “unstructured” and required a typical free-text answer whereas question 3 was “structured” and offered a selection of predefined wordings/phrases for participants to use in their free-text answer. The NLP engine was designed using word lists, rule-based synonyms, and decision tree learning based on the answer keys and its performance tested against the gold standard of reviewer markings.

**Results:**

After implementing the NLP approach in Python, F1 scores were calculated as a measure of NLP performance: 0.26 (unstructured question 1, *n* = 96), 0.33 (unstructured question 2, *n* = 327), and 0.5 (more structured question, *n* = 111). The respective precision/recall values were 0.26/0.27, 0.4/0.32, and 0.62/0.55.

**Conclusion:**

This study showed the successful design of an NLP-based approach for automatic evaluation of free-text answers in the EDiR examination. Thus, as a future field of application, NLP could work as a decision-support system for reviewers and support the design of examinations being adjusted to the requirements of an automated, NLP-based review process.

**Clinical relevance statement:**

Natural language processing can be successfully used to automatically evaluate free-text answers, performing better with more structured question-answer formats. Furthermore, this study provides a baseline for further work applying, e.g., more elaborated NLP approaches/large language models.

**Key points:**

• Free-text answers require manual evaluation, which is time-consuming and potentially error-prone.

• We developed a simple NLP-based approach — requiring only minimal effort/modeling — to automatically analyze and mark free-text answers.

• Our NLP engine has the potential to support the manual evaluation process.

• NLP performance is better on a more structured question-answer format.

**Graphical Abstract:**

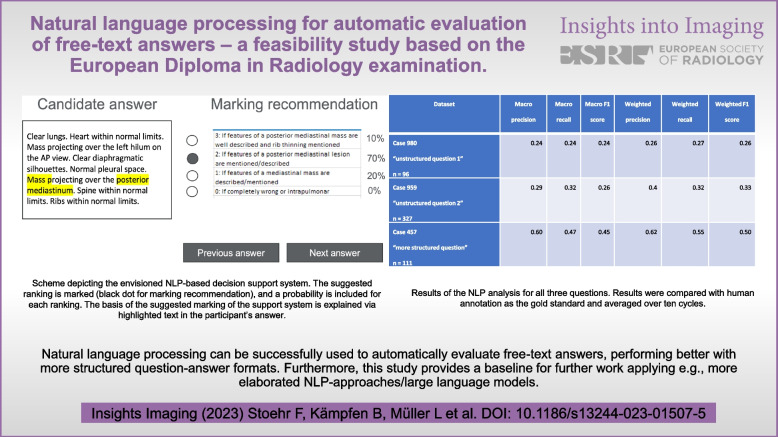

**Supplementary Information:**

The online version contains supplementary material available at 10.1186/s13244-023-01507-5.

## Background

A written examination is an established method for assessing performance [1, 2] and typically consists of multiple-choice questions (MCQs) and/or open-ended question formats, including free-text answers [1, 2]. MCQs are usually used in medical examinations, likely because they are more structured and allow for an easy-to-handle, objective, and cost-effective assessment [3–5].

Studies suggest, however, that open-ended question formats such as free-text responses are particularly well suited to promote a meaningful and sustainable learning process by requiring active recall of knowledge [6, 7]. A limitation on the resource side is that automatized assessment is often not feasible for these kinds of questions. Intensive manual evaluation is required, with human effort related to the need for expert knowledge in the field and a specific understanding of marking and grading criteria [8, 9]. Given the repetitive nature of the task, manual assessment also is highly time-consuming and potentially error-prone [8, 9].

Repetitive tasks, however, seem to be ideally suited for support from artificial intelligence, and natural language processing (NLP) offers a machine-based approach to automatically structure and analyze natural free text [10]. By applying various terms, synonyms, and language concepts, NLP transfers unstructured language information into a standardized form [10] that can be used to build a decision-support system [11]. Previous findings have indicated possible applications of NLP in various tasks ranging from clinical [12–14] to educational settings [8, 15–17].

Thus, we aimed to test whether NLP can be used to automatically analyze and code free-text answers to build a decision-support system for further enhancement of the review process. To this end, we conducted this feasibility study using old European Diploma in Radiology (EDiR) examinations. The EDiR examination is an additional qualification of excellence, and it serves as a tool for the standardization and accreditation of radiologists across European borders. It provides an international benchmark for general radiology and is officially and fully endorsed by the European Union of Medical Specialists (UEMS) and the European Society of Radiology (ESR).

We have chosen the EDiR examination because it is taken by a large number of radiologists every year, relies not only on highly standardized MCQs but intentionally also on open-ended question formats, and requires manual evaluation of free-text answers by several independent board-certified reviewers. With a substantial number of questions to evaluate, manually evaluating free-text answers can be overwhelming. Thus, there would be a huge potential to improve the review process, if an NLP approach would be applicable to the evaluation of free-text answers.

## Methods

### Study set-up

The European Board of Radiology (EBR) that is an initiative of the ESR provided representative datasets comprising cases with sample questions from the EDiR examination, corresponding answer keys from EBR together with original answers from the participants, and markings from the reviewers. Each case consists of one or more questions, and every question consists of a task description, a correct answer including an answer key, and a marking description. We used three questions from three different cases in this study: case 980, question 1 (unstructured question 1); case 959, question 1 (unstructured question 2); and case 457, question 1 (more structured question). Briefly, case 980 deals with an older fracture of the coronoid process, case 959 deals with prostatic lesions, and case 457 deals with a posterior mediastinal mass (see Supplementary Data for detailed descriptions of all three cases).

We chose the three questions based on the selection criteria of having the highest number of participant answers and being from a question pool consisting of so-called structured and unstructured questions. All three questions/cases required free-text responses, but case 457 was more “structured” than the other two because parts of the question included phrases that participants could consider using in their free-text answers. The other two cases required standard free-text responses with no phrasing options offered.

### Development of the NLP approach

**Fig. 1:**
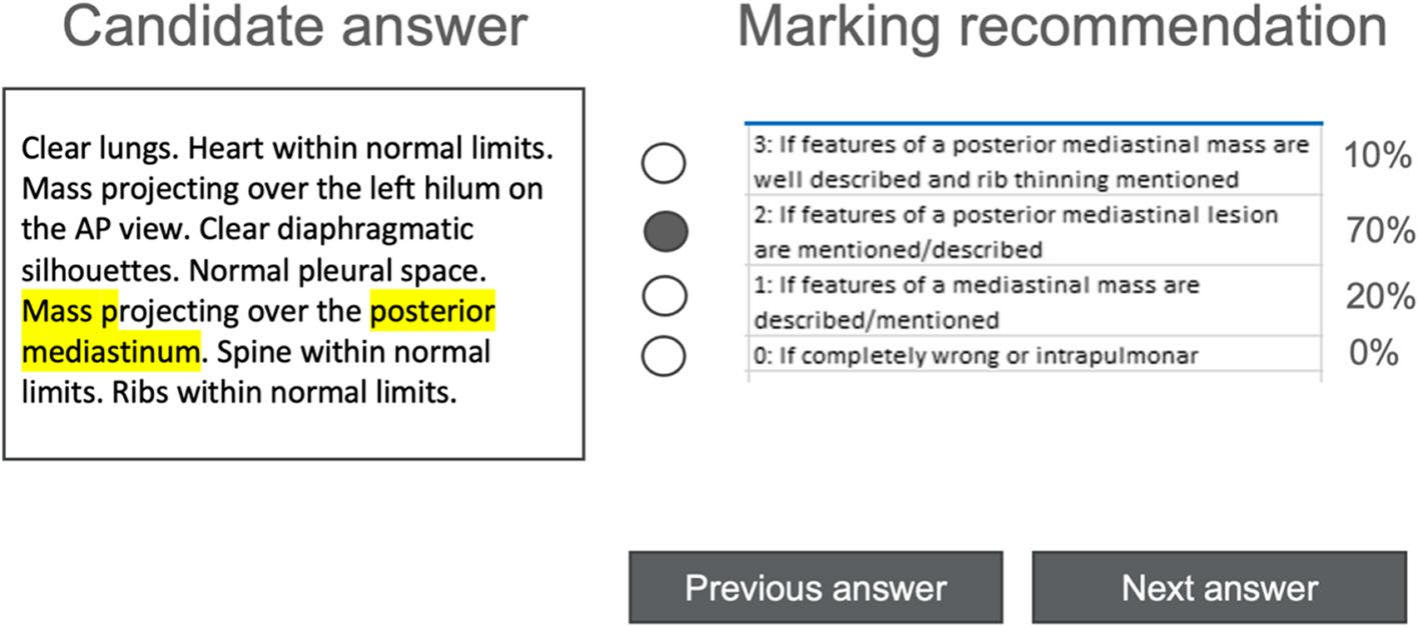
Scheme depicting the envisioned NLP-based decision support system. The suggested ranking is marked (black dot for marking recommendation), and a probability is included for each ranking. The basis of the suggested marking of the support system is explained via highlighted text in the participant’s answer

The NLP approach was planned to provide decision support for automatically evaluating and scoring free-text answers, clearly presenting data by highlighting and explaining responses, and calculating the results (0–4 points for case 959 and 0–3 points for case 980 and case 457, as predefined by the official answer key) as a proposal for rating the individual answers (Fig. 1).

**Fig. 2:**
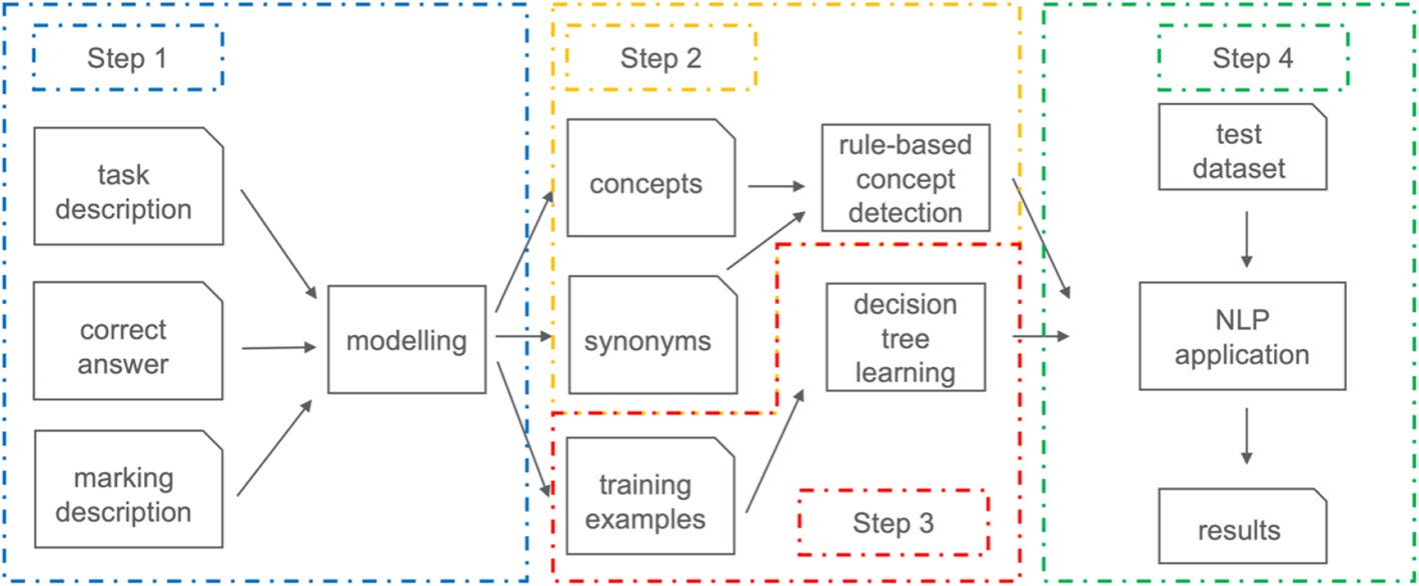
Process diagram illustrating our approach. Based on the task description, the correct answer and marking description concepts with synonyms and training examples are modeled (step 1 as described in the text, blue-dotted box). Based on concepts and synonyms, a rule-based concept detection is learned (step 2 as described in the text, yellow-dotted box), and at the same time, a decision tree is learned on the training examples (step 3 in the text, red-dotted box). Finally, the NLP can be run (step 4 in the text, green-dotted box)

For any subsequent use of the NLP engine in EDiR examinations, the effort required to describe the correct responses must be minimal, and no training data from previous examinations should be necessary since questions are changed regularly. For this task, the NLP approach had to be as simple as possible and thus was built on word lists, rule-based synonyms, and decision tree learning based on the official answer keys. The four steps of the selected approach are as follows (see also Fig. 2):

- *Step 1*: The NLP models concepts with synonyms and training examples based on the task description, the correct answer, and the marking description (provided by the EBR/the question submitter). For this task, concepts can be selected from open terminologies/specialized medical lexica such as RadLex®. However, concepts can also be “freely” selected (independent from such terminologies). Training examples included concepts assigned to official markings.
- *Step 2*: Based on the concepts and synonyms chosen, a rule-based concept detection is learned.
- *Step 3*: Based on the training examples, a decision tree is learned.
- *Step 4*: A test dataset is analyzed and evaluation metrices are computed.

### Building the NLP engine

As a proof of feasibility of our planned NLP approach, we used question 1 from case 980 (see Supplementary Data for details). As a first step, concepts and synonyms were selected based on the official answer key provided by EBR. For this task, RadLex® definitions were used for conceptualization. Furthermore, training examples were modeled based on the chosen concepts and their assigned marking. Marking definition was — again — provided by EBR.

To provide a better understanding of step 1 (modeling of concepts with synonyms and training examples), we have given excerpts of concepts with synonyms and training examples in Tables 1 and 2 (see Supplementary Tables 1 and 2 for the complete data).

**Table 1:** Excerpt of concepts with synonyms for case 980, question 1. RadLex® definitions were used for conceptualization

**Concept**	**Synonyms (examples)**
RID34330 persistent	Persistent Continual
RID45728 lucent	Lucent Transparent
RID34809 anatomical line of bone	Anatomical line of bone Line
RID2125 coronoid process of ulna	Coronoid process of ulna Processus coronoideus
RID4650 fracture	Fracture Lesion
RID4872 effusion	Effusion Fluid

**Table 2:** Excerpt of training examples with assigned markings for case 980, question 1

**Example**	**Marking**
RID45728 lucent	0
RID34330 persistent RID45728 lucent RID34809 anatomical line of bone RID2125 coronoid process of ulna	1
RID4650 fracture RID57221 old	1
RID34330 persistent RID45728 lucent RID34809 anatomical line of bone RID2125 coronoid process of ulna RID4805 bone fragment	2
RID34330 persistent RID45728 lucent RID34809 anatomical line of bone RID2125 coronoid process of ulna RID4872 effusion RID6122 joint RID4805 bone fragment RID5823 inferior RID1985 medial epicondyle of humerus RID2016 radiocapitellar joint RID39121 lateral	3

**Fig. 3:**
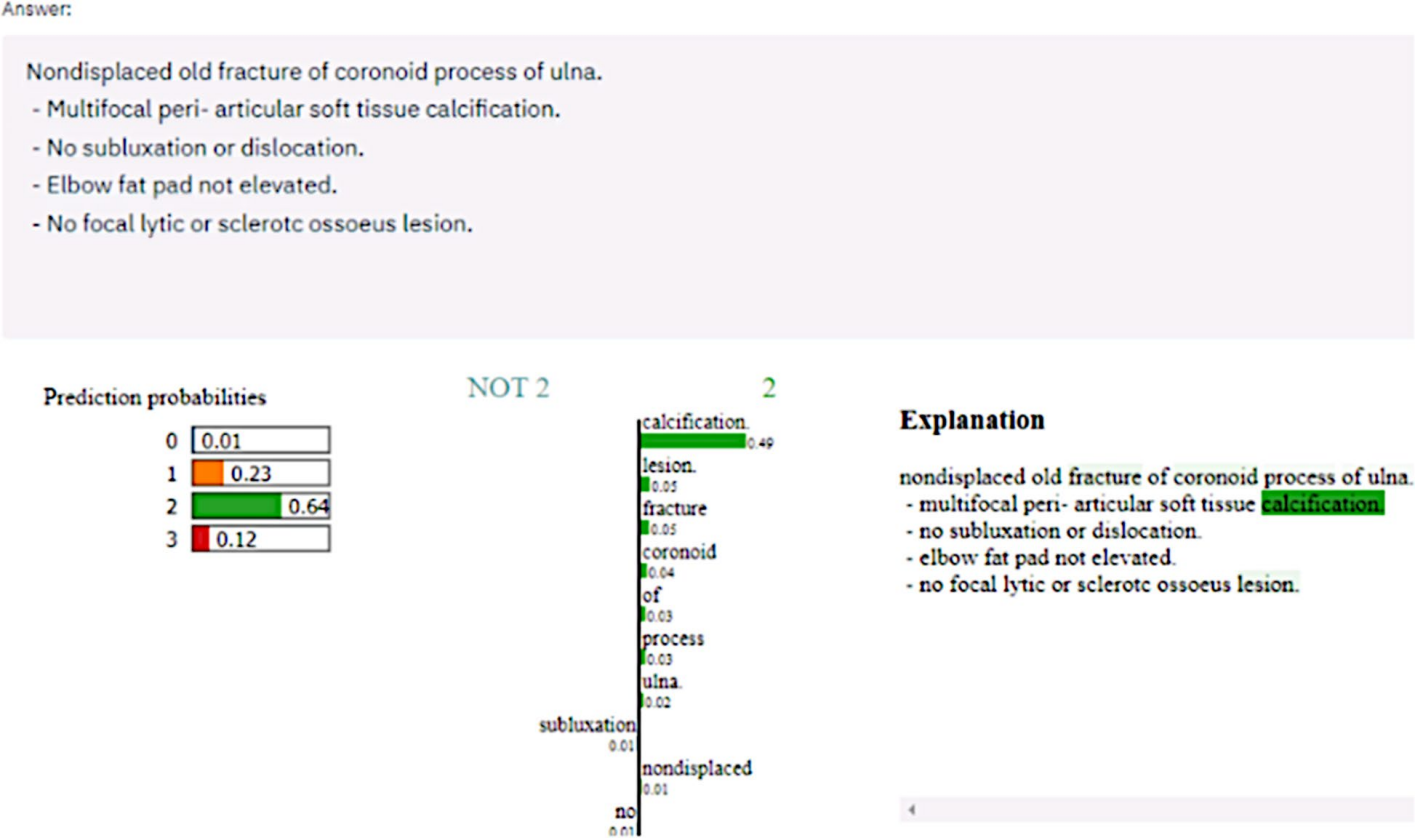
Interface for the NLP-based decision-support system for case 980, question 1. In the upper third (“answer”), the participant’s original answer can be seen (spelling errors have been left as in the original answer). On the left side (“prediction probabilities”), the suggested ranking is marked, including a probability for each suggestion given by the NLP. On the right side (“explanation”), the marking suggested by the NLP support system is explained (green highlighted text)

After performing steps 2 and 3 (see Fig. 2), the NLP approach for case 980, question 1 was implemented using Python, and a prototypical user interface using the Streamlit and Local Interpretable Model-agnostic Explanations (LIME) libraries was built (Fig. 3).

### Expanding the NLP approach

**Fig. 4:**
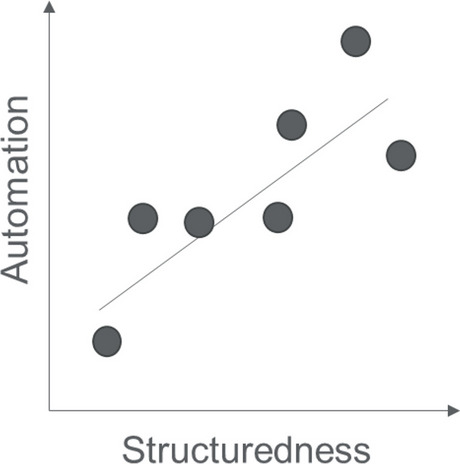
Pictorial presentation of our second hypothesis that the more structured a question–answer format is, the better the automation of this NLP approach will be because “automation” and “structuredness” must be seen as a spectrum

After running through the NLP process pipeline for case 980, we developed two hypotheses. First, despite the very simple NLP approach (rule-based concept detection and classification, without negation detection, without lemmatization, and without upfront learning on real examples), initial scores suggested better-than-random performance of the engine, which would show learning. Second, performance might be higher with more structured questions and answers compared with fully free-text responses because “automation” and “structuredness” can be seen as a spectrum, as depicted graphically in Fig. 4.

To test these hypotheses, we used two more cases and questions for the NLP approach (see the “Study set-up” section in the “Methods” section for details): the second “unstructured” case 959, question 1, and the “more structured” case 457, question 1. For these two new questions, we followed the same NLP approach as for case 980 and as depicted in Fig. 2. Concepts with synonyms and training examples for the two questions are illustrated in Supplementary Tables 3 and 4 (case 959) and Supplementary Tables 5 and 6 (case 457). Table 3 gives a summary of the number of concepts, synonyms, and training examples for each case, illustrating the pre-process human modeling effort required.

**Table 3:** Summary of the modeling for all three cases and questions regarding the total number of concepts, synonyms, and training examples

**Dataset**	**Number of concepts**	**Number of synonyms**	**Number of examples**
Case 980 “unstructured question 1”	16	89	34
Case 959 “unstructured question 2”	24	42	12
Case 457 “more structured question”	6	38	6

### NLP analysis

After modeling of the concepts, the synonyms, and the training examples and after learning a decision tree (steps 1–3 in Fig. 2), our NLP engine was used to analyze and mark answers from participants. The official answer key was provided by EBR and predefined the ranking (0–4 points for case 959 and 0–3 points for case 980 and case 457, as predefined by the official answer key).

## Results

In the following section, core results are presented in written form as well as listed in tabular form.

Results of the NLP were averaged over 10 cycles and compared with the gold standard (markings provided by the independent board-certified reviewers). Table 4 gives an overview of the cases.

**Table 4:** Number of cases and distribution of the markings provided by the independent board-certified reviewers (gold standard) for all three cases. Regarding case 959, 0–4 points could be achieved by the participants

**Dataset**	**Marking (0–3 and 0–4, respectively)**					**Total**
	**0**	**1**	**2**	**3**	**4**	
Case 980 “unstructured question 1”	13	37	21	25	NA	96
Case 959 “unstructured question 2”	14	112	90	80	31	327
Case 457 “more structured question”	38	37	30	6	NA	111

F1 scores were used as an overall measure of system performance [18].

For each case, Table 5 gives weighted and macro F1 scores as well as precision and recall values reflecting the performance of the NLP, along with the number of answers evaluated.

**Table 5:** Results of the NLP analysis for all three questions. Results were compared with the gold standard (markings provided by the independent board-certified reviewers) and averaged over ten cycles

**Dataset**	**Macro precision**	**Macro recall**	**Macro F1 score**	**Weighted precision**	**Weighted recall**	**Weighted F1 score**
Case 980 “unstructured question 1” *n* = 96	0.24	0.24	0.24	0.26	0.27	0.26
Case 959 “unstructured question 2” *n* = 327	0.29	0.32	0.26	0.4	0.32	0.33
Case 457 “more structured question” *n* = 111	0.60	0.47	0.45	0.62	0.55	0.50

The highest F1 scores were obtained with case 457, question 1, the more structured of the three, with a weighted F1 score of 50% that clearly indicated a higher performance compared with the unstructured questions. Precision and recall, as would be expected, also were highest with this more structured case.

Performance with case 980, unstructured question 1, was a weighted F1 score below one-third (26%), possibly because of the small size of the test dataset.

For case 959, unstructured question 2, the weighted F1 score was 33%, low but still clearly above the baseline of random guessing. As the slightly higher macro versus weighted F1 scores in all cases indicate (Table 5), the NLP did not perform equally well across all markings.

## Discussion

This feasibility study showed that our NLP approach can be successfully used to automatically analyze and mark free-text answers. This offers the chance to automate the time-consuming and potentially error-prone manual evaluation process of human reviewers. We found that the NLP engine performed better with the more structured question-answer format tested here, likely because this format required significantly less effort for the NLP engine to function sufficiently.

To date, the best question type for assessing learning performance is still unclear [1]. At best, exam questions not only evaluate knowledge but also further enhance a sustainable and effective learning process [19]. Ideally, the assessment sharpens the knowledge and skills that are needed in a future workplace [19, 20]. For radiology, these requirements include medical expert knowledge and skills in critical thinking, problem-solving, patient care, and interpersonal communication [19, 21]. Studies suggest that open-ended question formats, such as free-text responses, are particularly suitable for confirming the acquisition of such skills because of their requirement for active application of understanding [22, 23]. However, the assessment of free-text answers demands high human effort in terms of considerable expert knowledge and time needed for reviewing [8, 9].

For the current study, we built the NLP to automatically analyze free-text answers as support for the review process. Despite our promising results, various factors must be treated with caution in this approach. As a first step, terms and synonyms must be selected based on the official answer key. Depending on the complexity of the question and answer, choosing the most fitting terms requires expert radiological knowledge and is accordingly time-consuming. For support in this task, specialized medical lexica (e.g., RadLex®) are available containing various medical definitions, including synonyms and terms. However, these sources sometimes do not provide the most suitable terms required for a question, so that relationships between medical terms are obscured or a medical term might be taken on multiple meanings. As an example, when the correct answer to a particular question is “ulna, radial and humerus fracture,” this specific term is not provided in these sources as a complete phrase but instead is fragmented as “ulna,” “radial,” “humerus,” and “fracture.” In this way, the terms limit the accuracy and reliability of the NLP engine because the parts do not reflect the unique meaning of the whole phrase.

Furthermore, non-standardized vocabulary can affect NLP accuracy. Radiological language is special in often involving the use of subjective phrases without formal consensus on meaning or impact [24]. Lee et al. showed how differently radiologists and non-radiologists interpret and use some supposedly clear phrases, implying an inconsistent use of language [25]. One solution could be to include only a highly restricted vocabulary, as Jungmann et al. successfully did with only a limited number of medical terms for NLP-based extraction of epidemiological information from radiological reports [12]. However, the free-text answers in the current work consisted of a variety of medical terms requiring an adjusted approach to meet marking needs. A more practical strategy could be to pre-define the radiological vocabulary required for the particular question/answer and strictly avoid ambiguous terms [25]. One example from clinical routine is the use of vocabulary from standardized reporting systems (e.g., Coronary Artery Disease–Reporting and Data System (CAD-RADS) or Liver Imaging-Reporting and Data System (LI-RADS)), which was implemented to minimize language variations and ambiguity in terminology [26]. A closer look at cases 980 and 959 in the current study suggests a related possible explanation for the better performance of the NLP engine with case 959, even though both cases were unstructured. In case 959, an intrinsically more standardized vocabulary from the Prostate Imaging-Reporting and Data System (PI-RADS) was used (PI-RADS version 2.1) [27].

Refining the spectrum of “automation” and “structuredness” to include “more structured” could lead to the question/answer format presented in case 457. In this case, participants had to give free-text answers but could consider a selection of predefined terms for their answers. Among the three cases, our NLP engine also had the best performance with this case involving the least “human effort” (number of concepts/synonyms and training examples used). These results are in line with inputs from clinical radiology suggesting that structured information (e.g., as so-called structured reports) could offer a benefit when it comes to mining data for relevant information [26, 28]. As suggested in our study, this benefit might arise from standardized report content and consistent language [26, 28].

Taken together, our findings suggest that a standardized, structured question calling for a clear answer with a highly specific and standardized vocabulary would allow for the most effective NLP approach. However, the more structured a question-answer format is, the more it might be comparable to an MCQ format, which no longer requires NLP methods for assessment. To summarize, the results of this study illustrate a possible spectrum of structure needed in question-answer formats on which an NLP engine can work sufficiently.

### Limitations and future work

This study has several limitations. First, the content recognition of the NLP engine used in this work is not perfect, in part likely because of the long and “creative” phrasing of questions and answer keys. Often, this feature hampered the choice of the most fitting terms that would have allowed for sufficient NLP performance. As noted, using standardized and unambiguous vocabulary when designing questions and answer keys would probably allow for better NLP performance.

Furthermore, questions are not reused in the same exact layout by EBR, which along with the rather small sample size could limit training data for machine learning. Thus, in this feasibility study, we used NLP approaches that required no upfront learning on training data but that could be modeled easily based on available information about the correct answer. However, more training data might improve the NLP engine by allowing for “more sophisticated” approaches. Taking the small sample size of three questions into account, a general transferability of our approach into daily routine is questionable. However, future studies could build on our proof-of-concept study and could use it for further development of similar approaches.

Last, inherently due to the design of our study as a proof-of-concept work, we did not investigate to what extent our NLP approach leads, e.g., to a shortened review time or even better review results in daily practice. For this, further studies would be necessary and could investigate if NLP approaches affect inter-reviewer variability or save time for the reviewers.

These ideas/further studies could be combined with the use of “more elaborate”.

NLP approaches such as active learning systems that would self-update after each marking or large language models like, e.g., ChatGPT [29]. On the one hand, large language models are well applicable since questions and correct answer keys are available and can be used as prompts for such generative artificial intelligence methods. However, automatic evaluation of free-text answers will remain difficult: due to spelling errors, due to a highly implicit language in both free-text answers and questions and correct answer keys, and due to a limited amount of specific training data as questions are changed regularly as part of the examination process.

Additionally, there are potential inherent disadvantages of large language models such as unforeseen bias from the training text corpus, their black box nature with difficulties explaining their reasoning, large computing power needed for training and/or finetuning, and their usage with possibly sensitive data on restricted third-party platforms.

To these ends, this study using comprehensible and reproducible approaches provides a baseline for further work on this topic.

## Conclusion

NLP can be successfully used to automatically evaluate free-text answers and showed promising results in three examples cases of the EDiR examination. Considering a spectrum of “automation” of the review process and “structuredness” of the question-answer format, our NLP approach performed better on a more structured question-answer format that still retained free-text elements. In summary, this study provides a baseline for further work applying more elaborate NLP approaches, in particular, large language models for automatic evaluation of free-text answers. With this in mind, NLP could kill two birds with one stone: by working as a decision-support system to improve the review process and by supporting the design of examinations that are better adjusted to the requirements of NLP-based, automated analysis and marking.

### Supplementary Information

**Additional file 1.**

## Data Availability

All data generated or analyzed during this study are included in this published article (and its supplementary information files).

## Abbreviations

CAD-RADS: Coronary Artery Disease–Reporting and Data System
EBR: European Board of Radiology
EDiR: European Diploma in Radiology
ESR: European Society of Radiology
LI-RADS: Liver Imaging-Reporting and Data System
LIME: Local Interpretable Model-agnostic Explanations
MCQ: Multiple-choice questions
NLP: Natural language processing
PI-RADS: Prostate Imaging-Reporting and Data System
UEMS: European Union of Medical Specialists
