# Supplementary material for: Natural language processing for automatic evaluation of free-text answers — a feasibility study based on the European Diploma in Radiology examination
Source: Insights Imaging. 2023 Sep 19;14:150. doi: 10.1186/s13244-023-01507-5 (PMC10509084; doi:10.1186/s13244-023-01507-5)
Supplement: Supplementary file 1 — Additional file 1. [file 13244_2023_1507_MOESM1_ESM.docx]

**Natural language processing for automatic evaluation of free-text answers – a feasibility study based on the European Diploma in Radiology examination**

**ELECTRONIC SUPPLEMENTARY MATERIAL**

**Supplementary data case 980.** Complete question/answer format including the original question from EBR, answer key with correct answer and marking description

**Supplementary data case 959.** Complete question/answer format including the original question from EBR, answer key with correct answer and marking description

**Supplementary data case 457.** Complete question/answer format including the original question from EBR, answer key with correct answer and marking description


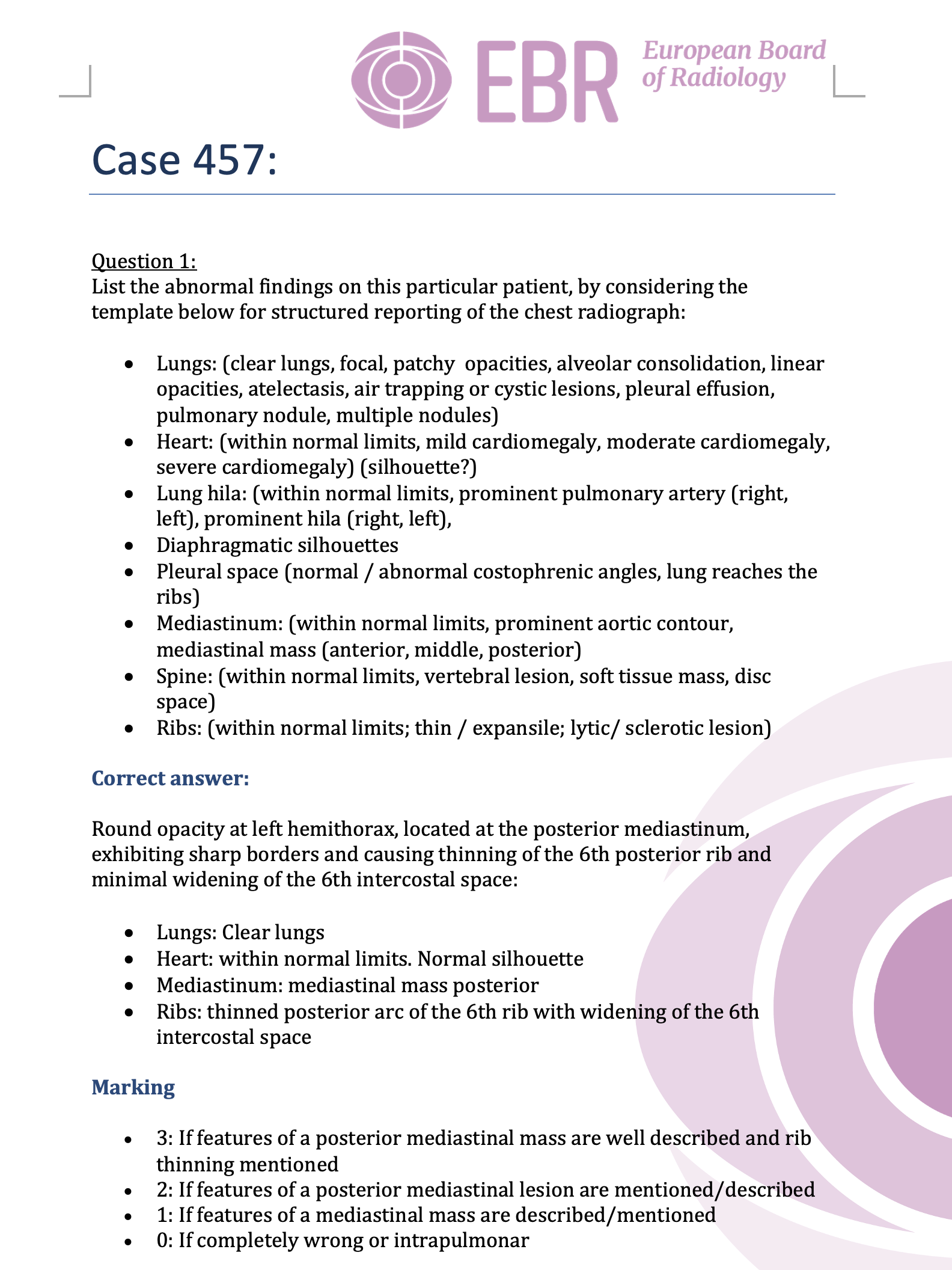


**Supplementary Table 1.** Concepts with synonyms for case 980 question 1.

| **Concept** | **Synonyms** |
| --- | --- |
| RID34330persistent | persistent  continual  pers  persitency  persisting |
| RID45728lucent | lucent  lucency  transparent |
| RID34809anatomicallineofbone | anatomical line of bone  line  linear |
| RID2125coronoidprocessofulna | coronoid process of ulna  processus coronoideus  process |
| RID4650fracture | fracture  pseudarthrosis  lesion  impaction  fractured  fissure  interruption  bone fragment  flake  osseous flake  osseous fragment  calcification  bony fragment  calcified body  density  bony fragmentations  ossification  bony body |
| RID57221old | old  not healed  sclerotic  non-unioned  non-union  unconsolidated  incomplete healing |
| RID4872effusion | effusion  fluid  hydrops |
| RID6122joint | joint  intraarticular  articular |
| RID4805bonefragment | bone fragment  flake  osseous flake  osseous fragment  calcification  bony fragment  calcified body  density  bony fragmentations  ossification  bony body  fracture  pseudarthrosis  lesion  impaction  fractured  fissure  interruption |
| RID1985medialepicondyleofhumerus | medial epicondyle of humerus  medial humeral epicondyle  medial epicondyle |
| RID2016radiocapitellarjoint | radiocapitellar joint  humeroradial joint  radiohumeral joint |
| RID4751displacement | displacement  dislocation  dislocated  displaced  elevation  sail sign  bulging  swelling  shadow  uplifting  uplifted  positive |
| RID35202fatpadsign | fat pad sign  fat pad  sail sign  fat planes  edema |
| RID5765multiple | multiple |
| RID5823inferior | inferior |
| RID39121lateral | lateral |

**Supplementary Table 2.** Training examples including markings for case 980 question 1.

| **Answer** | **Marking** |
| --- | --- |
|  | 0 |
| RID34330persistent | 0 |
| RID45728lucent | 0 |
| RID34809anatomicallineofbone | 0 |
| RID2125coronoidprocessofulna | 0 |
| RID57221old | 0 |
| RID4872effusion | 0 |
| RID6122joint | 0 |
| RID35202fatpadsign | 0 |
| RID4751displacement | 0 |
| RID5765multiple | 0 |
| RID5823inferior | 0 |
| RID1985medialepicondyleofhumerus | 0 |
| RID2016radiocapitellarjoint | 0 |
| RID39121lateral | 0 |
| RID4650fracture | 0 |
| RID34330persistent RID45728lucent RID34809anatomicallineofbone RID2125coronoidprocessofulna | 1 |
| RID4650fracture RID57221old | 1 |
| RID4805bonefragment | 1 |
| RID4650fracture RID4805bonefragment | 1 |
| RID34330persistent RID45728lucent RID34809anatomicallineofbone RID2125coronoidprocessofulna RID4805bonefragment | 2 |
| RID4650fracture RID57221old RID4805bonefragment | 2 |
| RID34330persistent RID45728lucent RID34809anatomicallineofbone RID2125coronoidprocessofulna RID4872effusion RID6122joint RID4805bonefragment RID5823inferior RID1985medialepicondyleofhumerus | 3 |
| RID2125coronoidprocessofulna RID4650fracture RID57221old RID4872effusion RID6122joint RID4805bonefragment RID5823inferior RID1985medialepicondyleofhumerus | 3 |
| RID34330persistent RID45728lucent RID34809anatomicallineofbone RID2125coronoidprocessofulna RID4872effusion RID6122joint RID4805bonefragment RID2016radiocapitellarjoint RID39121lateral | 3 |
| RID2125coronoidprocessofulna RID4650fracture RID57221old RID4872effusion RID6122joint RID4805bonefragment RID2016radiocapitellarjoint RID39121lateral | 3 |
| RID34330persistent RID45728lucent RID34809anatomicallineofbone RID2125coronoidprocessofulna RID4872effusion RID6122joint RID4805bonefragment RID5823inferior RID1985medialepicondyleofhumerus RID2016radiocapitellarjoint RID39121lateral | 3 |
| RID4650fracture RID57221old RID4872effusion RID6122joint RID4805bonefragment RID5823inferior RID1985medialepicondyleofhumerus RID2016radiocapitellarjoint RID39121lateral | 3 |
| RID34330persistent RID45728lucent RID34809anatomicallineofbone RID2125coronoidprocessofulna RID35202fatpadsign RID4751displacement RID4805bonefragment RID5823inferior RID1985medialepicondyleofhumerus | 3 |
| RID2125coronoidprocessofulna RID4650fracture RID57221old RID35202fatpadsign RID4751displacement RID4805bonefragment RID5823inferior RID1985medialepicondyleofhumerus | 3 |
| RID34330persistent RID45728lucent RID34809anatomicallineofbone RID2125coronoidprocessofulna RID35202fatpadsign RID4751displacement RID4805bonefragment RID2016radiocapitellarjoint RID39121lateral | 3 |
| RID2125coronoidprocessofulna RID4650fracture RID57221old RID35202fatpadsign RID4751displacement RID4805bonefragment RID2016radiocapitellarjoint RID39121lateral | 3 |
| RID2125coronoidprocessofulna RID35202fatpadsign RID4751displacement RID4805bonefragment RID5823inferior RID1985medialepicondyleofhumerus RID2016radiocapitellarjoint RID39121lateral | 3 |
| RID4650fracture RID57221old RID35202fatpadsign RID4751displacement RID4805bonefragment RID5823inferior RID1985medialepicondyleofhumerus RID2016radiocapitellarjoint RID39121lateral | 3 |

**Supplementary Table 3**. Concepts with synonyms for case 959 question 1.

| **Code** | **Synonyms** |
| --- | --- |
| RIDE_base | base  bottom |
| RIDE_behavior | behavior  behaviour |
| RIDE_enlarged | enlarged  enlargement |
| RIDE_gland | gland |
| RIDE_homogenous | homogenous |
| RIDE_hypointense | hypointense  hypointensity  low signal intensity |
| RIDE_infiltration | infiltration  extension  invasion  involvement  growth |
| RIDE_invasive | invasive |
| RIDE_lesion | lesion  nodule  area  lesions |
| RIDE_mid | mid  mid-portioned  mid-portion |
| RIDE_moderately | moderately  relatively  mild |
| RIDE_peripheral | peripheral |
| RIDE_prostate | prostate |
| RIDE_transition | transition |

**Supplementary Table 4.** Training examples with markings for case 959 question 1:

| **Answer** | **Marking** |
| --- | --- |
|  | 0 |
| RIDE_lesion | 1 |
| RIDE_lesion RIDE_transition RIDE_peripheral | 2 |
| RIDE_lesion RIDE_mid RIDE_base | 2 |
| RIDE_lesion RIDE_transition RIDE_peripheral RIDE_invasive  RIDE_behavior | 3 |
| RIDE_lesion RIDE_mid  RIDE_base RIDE_invasive  RIDE_behavior | 3 |
| RIDE_lesion RIDE_transition  RIDE_peripheral  RIDE_infiltration | 3 |
| RIDE_lesion RIDE_mid  RIDE_base RIDE_infiltration | 3 |
| RIDE_lesion RIDE_transition RIDE_peripheral  RIDE_invasive  RIDE_behavior RIDE_gland RIDE_enlarged RIDE_homogeneous RIDE_moderately RIDE_hypointense | 4 |
| RIDE_lesion RIDE_mid RIDE_base RIDE_invasive  RIDE_behavior RIDE_gland RIDE_enlarged RIDE_homogeneous RIDE_moderately RIDE_hypointense | 4 |
| RIDE_lesion RIDE_transition RIDE_peripheral RIDE_infiltration RIDE_gland RIDE_enlarged RIDE_homogeneous RIDE_moderately RIDE_hypointense | 4 |
| RIDE_lesion RIDE_mid RIDE_base RIDE_infiltration RIDE_gland RIDE_enlarged RIDE_homogeneous RIDE_moderately RIDE_hypointense | 4 |

**Supplementary Table 5.** Concepts with synonyms for case 457 question 1.

| **Code** | **Synonyms** |
| --- | --- |
| RIDE_Lungs_Clear_lungs | Lungs: Clear lungs |
| RIDE_Heart_within_normal_limits | Heart: within normal limits |
| RIDE_Mediastinum_mediastinal_mass_posterior | Mediastinum: mediastinal mass posterior  mediastinal mass posterior  posterior mediastinal mass  mediastinal mass posterior  mediastinal lesion posterior  mediastinal consolidation posterior  mediastinal density posterior  mediastinal nodule posterior  mediastinal opacification posterior  mediastinal opacity posterior  mediastinal increased opacity posterior  posterior mediastinal mass  posterior mediastinal lesion  posterior mediastinal consolidation  posterior mediastinal density  posterior mediastinal nodule  posterior mediastinal opacification  posterior mediastinal opacity  posterior mediastinal increased opacity |
| RIDE_Ribs_thin | Ribs: thin  rib thinning  rib lytic  Ribs: lytic  thinning of the rib  thinning of costa |
| RIDE_Mediastinum_mediastinal_mass | Mediastinum: mediastinal mass  mediastinal mass  mediastinal lesion  mediastinal consolidation  mediastinal density  mediastinal nodule  mediastinal opacification  mediastinal opacity  mediastinal increased opacity |
| RIDE_intrapulmonal | intrapulmonal |

**Supplementary Table 6.** Training examples for case 457 question 1.

| **Answer** | **Marking** |
| --- | --- |
| RIDE_Mediastinum_mediastinal_mass RIDE_Mediastinum_mediastinal_mass_posterior RIDE_Ribs_thin | 3 |
| RIDE_Mediastinum_mediastinal_mass RIDE_Mediastinum_mediastinal_mass_posterior | 2 |
| RIDE_Mediastinum_mediastinal_mass | 1 |
| RIDE_intrapulmonar | 0 |
| RIDE_Ribs_thin | 1 |
|  | 0 |
